# Downregulation of cGAS/STING expression in tumor cells by cancer-associated fibroblasts in colorectal cancer

**DOI:** 10.1038/s41598-025-03924-6

**Published:** 2025-06-02

**Authors:** Ryo Kanoda, Shotaro Nakajima, Hirokazu Okayama, Akinao Kaneta, Shun Chida, Takuro Matsumoto, Katsuharu Saito, Tomohiro Kikuchi, Yuya Maruyama, Hiroya Suzuki, Kosaku Mimura, Motonobu Saito, Hiroyuki Hanayama, Wataru Sakamoto, Tomoyuki Momma, Zenichiro Saze, Koji Kono

**Affiliations:** 1 Department of Gastrointestinal Tract Surgery, School of Medicine, Fukushima Medical University, Fukushima, Japan; 2 Department of Multidisciplinary Treatment of Cancer and Regional Medical Support, School of Medicine, Fukushima Medical University, 1 Hikariga-oka, Fukushima, Fukushima 960-1295 Japan; 3 Department of Blood Transfusion and Transplantation Immunology, School of Medicine, Fukushima Medical University, Fukushima, Japan

**Keywords:** Colorectal cancer, Cancer-associated fibroblast, Versican, Tumor cell-intrinsic cGAS–STING, Colorectal cancer, Cancer microenvironment

## Abstract

The tumor cell-intrinsic cyclic GMP–AMP synthase (cGAS)–stimulator of interferon genes (STING) pathway is critical for activating anti-tumor immunity and enhancing immune checkpoint blockade therapy in colorectal cancer (CRC). Cancer-associated fibroblasts (CAFs), key components of the CRC tumor microenvironment, negatively regulate the anti-tumor immune response. However, their impact on tumor cell-intrinsic cGAS–STING expression remains unclear. In the present study, we investigated whether CAFs can downregulate cGAS–STING expression in CRC. We found that cGAS–STING expression in tumor cells inversely correlated with stromal expression of versican (VCAN), an immunosuppressive CAF marker, in CRC tissues. Co-culture experiments using primary human CAFs derived from CRC tissues revealed that CAFs downregulated cGAS and/or STING expression in CRC cell lines (WiDr, LoVo, HCT116). Furthermore, CAFs expressing *VCAN* and *fibronectin 1* appeared to mediate this suppression. These findings suggest that immunosuppressive CAFs contribute to the downregulation of tumor cell-intrinsic cGAS–STING expression in CRC. Therefore, targeting CAFs to restore cGAS–STING expression may represent a promising strategy to enhance the efficacy of CRC treatment.

## Introduction

Colorectal cancer (CRC) is the most common type of gastrointestinal cancer and the second leading cause of cancer-related deaths worldwide^1^. Over the past decade, therapeutic strategies for CRC have been developed and personalized to address genetic alterations such as *RAS* and *BRAF* mutations, as well as molecular features such as microsatellite instability, which can result from both genetic and epigenetic alterations, leading to improved patient outcomes^2–4^. However, these targeted therapies are only available to a small subset of patients, and advanced-stage CRC still has a poor prognosis. Therefore, further development of therapeutic strategies is crucial for the prognosis of CRC.

The cyclic GMP–AMP synthase (cGAS)–stimulator of interferon genes (STING) pathway plays a crucial role in activating inflammation and the type I interferon (IFN) response against microbial and host-derived double-stranded DNA caused by infection, cellular stress, and DNA damage within the cytoplasm of mammalian cells^5–8^. Activation of the cGAS–STING pathway phosphorylates downstream transcription factors, including IFN regulatory factor 3 and nuclear factor-κB, leading to the production of type I IFN, inflammatory cytokines, and T-cell-attracting chemokines^6,8,9^. Notably, the tumor cell-intrinsic cGAS–STING pathway plays a critical role in anti-tumor immunity, and has been shown to enhance the efficacy of cancer immunotherapy in several cancers, including CRC^8,10–13^. Chon et al. reported that CRC patients with high tumor cell-intrinsic STING expression exhibited increased CD8^+^ tumor-infiltrating lymphocytes (TILs) and better clinical outcomes compared to those with low tumor cell-intrinsic STING expression^14^. Furthermore, our previous study and others have reported that tumor cell-intrinsic cGAS–STING expression was decreased in CRC, particularly in advanced cases, and this suppression was significantly associated with a lower frequency of CD8^+^ TILs, poor patient prognosis, and poor response to immune checkpoint blockade (ICB) therapy^15–17^. Additionally, we previously reported that tumor cell-intrinsic cGAS–STING expression was significantly higher in mismatch repair-deficient (dMMR) CRC compared to MMR-proficient (pMMR) CRC. This elevated expression contributes to the high infiltration of CD8^+^ T cells in the tumor microenvironment (TME) of dMMR CRC^18^. However, the mechanisms underlying the downregulation of tumor cell-intrinsic cGAS–STING expression in CRC remain poorly understood.

Cancer-associated fibroblasts (CAFs) are a central component of the TME and exhibit significant heterogeneity in their origins, phenotypes, and functions^19^. CAFs have been reported to play diverse roles in the progression of solid tumors including CRC, such as promoting cancer cell growth, angiogenesis, and metastasis, as well as remodeling the extracellular matrix, and these effects are induced by several mediators, including interleukin 6, vascular endothelial growth factor, transforming growth factor-β (TGF-β), and matrix metalloproteinases^19–23^. CAFs also play a crucial role in suppressing anti-tumor immune responses by fostering an immunosuppressive TME^24^, and a reduction in CD8^+^ T-cell infiltration has been observed in CAF-rich TMEs^25,26^. Regarding the mechanistic insight underlying CAF-mediated suppression of anti-tumor immune responses in CRC, CAFs recruit, differentiate, and activate immune cells involved in the inhibition of anti-tumor immunity, including M2-tumor-associated macrophages (M2-TAMs) and regulatory T cells, while excluding cytotoxic CD8^+^ T cells in the TME^27^. Additionally, CAFs promote immune evasion in CRC by upregulating PD-L1 expression in tumor cells^28^. Therefore, CAFs affect both immune cells and tumor cells negatively, suppressing the anti-tumor immune response in the CRC TME. We speculated that one potential mechanism contributing to CAF-mediated suppression of anti-tumor immune responses in CRC is the downregulation of tumor cell-intrinsic cGAS–STING expression.

In the present study, we aimed to investigate the impact of CAFs on tumor cell-intrinsic cGAS–STING expression in CRC, by analyzing the correlation between tumor cell-intrinsic cGAS–STING expression and the stromal expression of versican (VCAN), an immunosuppressive CAF-specific marker in CRC^29,30^. Additionally, we established primary CAFs from CRC tissues and co-cultured them with CRC cell lines in vitro to evaluate the effect of CAFs on cGAS–STING expression in CRC cells.

## Results

### Inverse correlation between stromal VCAN expression and tumor cell-intrinsic cGAS–STING expression in CRC

**Fig. 1:**
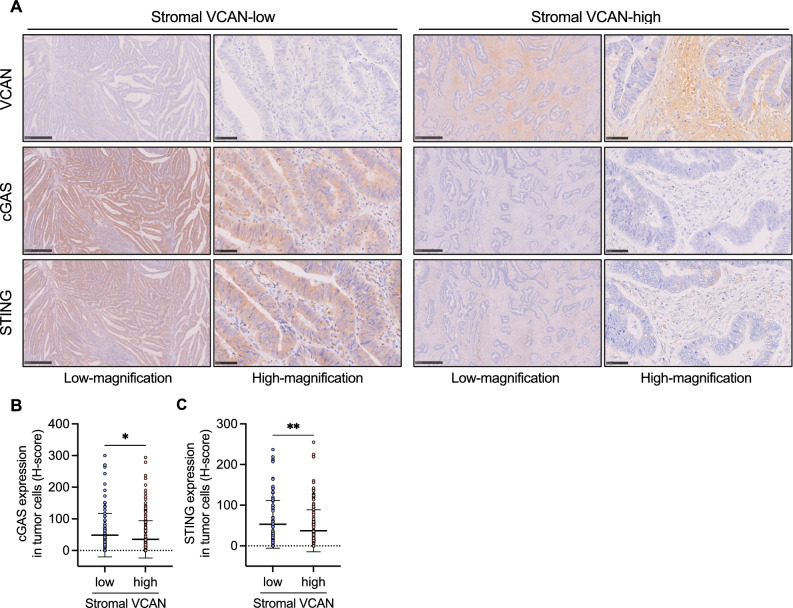
Association between stromal VCAN expression and tumor cell-intrinsic cGAS–STING expression in CRC. (**A**) Representative IHC images of VCAN, cGAS, and STING in surgically resected CRC specimens. Scale bars: 500 µm for low-magnification images and 100 µm for high-magnification images. (**B, C**) Comparison of H-scores for cGAS (**B**) and STING (**C**) between stromal VCAN-high and VCAN-low CRC groups. Values are presented as mean ± SD. Statistical significance was determined using the Mann–Whitney *U* test. *p < 0.05, **p < 0.01.

We first assessed the association between stromal VCAN expression and tumor cell-intrinsic cGAS–STING expression in CRC. Patients in the Fukushima Medical University (FMU) Cohort 1 were divided into stromal VCAN-high [VCAN immunohistochemistry (IHC) score ≥ 3, *n* = 161] and stromal VCAN-low (VCAN IHC score < 3, *n* = 107) groups based on their VCAN IHC score (Table 1). Consistent with our previous report^29^, stromal VCAN expression was higher in advanced stages compared to early stages of CRC (Table 1). cGAS and STING expression was assessed in tumor cells, independent of expression in the stromal area, using the IHC score (H-score) as described in *Methods* section. Representative IHC images of VCAN, cGAS, and STING for the stromal VCAN-high and -low groups are shown in Fig. 1A. Tumor cell-intrinsic cGAS and STING expression levels were significantly lower in the stromal VCAN-high group compared to the stromal VCAN-low group (Fig. 1B, C), suggesting a potential inverse correlation between stromal VCAN expression and tumor cell-intrinsic cGAS–STING expression in CRC. This indicates that immunosuppressive CAF-rich CRC might exhibit lower expression of tumor cell-intrinsic cGAS–STING.

**Table 1:** Clinicopathological characteristics of colorectal cancer patients according to stromal VCAN expression status (FMU cohort 1).

		VCAN-high (*n* = 161)	VCAN-low (*n* = 107)	*p* value
Age	Mean ± SD	67.6 ± 12.7	69.5 ± 12.3	0.1986
Sex				0.4455
	Male	100 (62.1%)	61 (57.0%)	
	Female	61 (37.9%)	46 (43.0%)	
Location				0.0475
	Proximal colon	52 (32.3%)	50 (46.7%)	
	Distal colon	42(26.1%)	25 (23.4%)	
	Rectum	67 (41.6%)	32 (29.9%)	
Differentiation				0.0301
	Well	67 (41.6%)	51 (47.7%)	
	Moderately	87 (54.0%)	44 (41.1%)	
	Poorly	7 (4.4%)	12 (11.2%)	
Tumor invasion				< 0.0001
	Tis	0 (0%)	13 (12.2%)	
	T1	7 (4.4%)	21 (19.6%)	
	T2	26 (16.1%)	17 (15.9%)	
	T3	77 (47.8%)	41 (38.3%)	
	T4	51 (31.7%)	15 (14.0%)	
Lymph node metastasis				< 0.0001
	Absent	79 (49.1%)	84 (78.5%)	
	Present	81 (50.3%)	23 (21.5%)	
	Not available	1 (0.6%)	0 (0.0%)	
Distant metastasis				0.0136
	Absent	138 (85.7%)	102 (95.3%)	
	Present	23 (14.3%)	5 (4.7%)	
Stage				< 0.0001
	0	0 (0.0%)	13 (12.2%)	
	I	22 (13.7%)	34 (31.8%)	
	II	54 (33.5%)	36 (33.6%)	
	III	61 (37.9%)	19 (17.7%)	
	IV	24 (14.9%)	5 (4.7%)	
MMR status				0.0832
	pMMR	142 (88.2%)	86 (80.4%)	
	dMMR	19 (11.8%)	21 (19.6%)	

*MMR* mismatch repair, *dMMR* MMR-deficient, *pMMR* MMR-proficient, *SD* standard deviation, *Tis* carcinoma in situ, *VCAN* versican.

### Effect of CAFs on tumor cell-intrinsic cGAS–STING expression in CRC

**Fig. 2:**
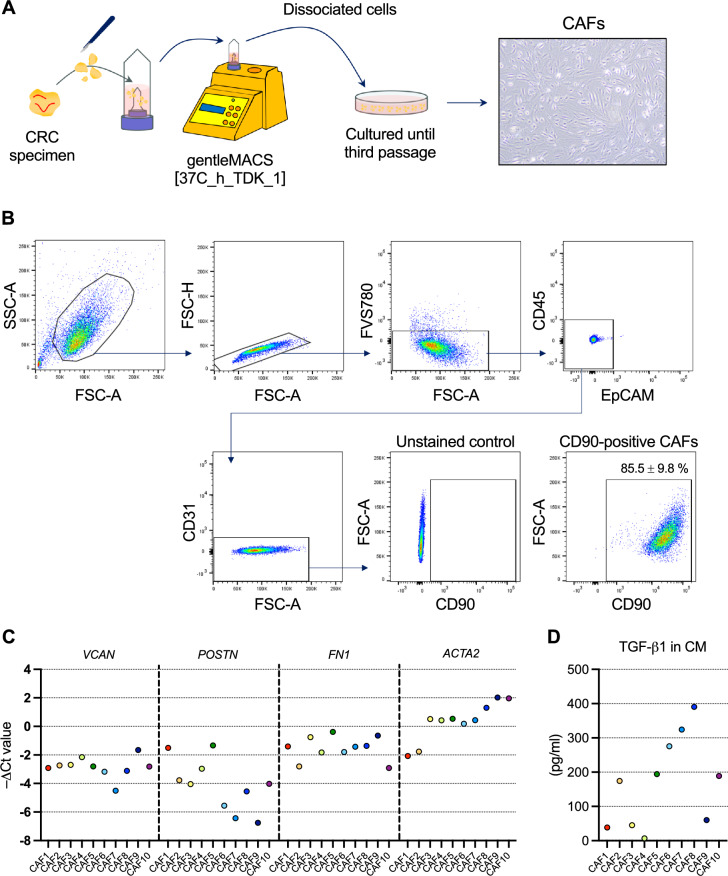
Isolation and characterization of human primary CAFs from CRC tissues. (**A**) Schema of CAF isolation and image of isolated primary CAFs. (**B**) Flow cytometry gating strategy to define human primary CAFs isolated from CRC tissues. (**C**) qPCR analysis of the indicated molecules in all cultured CAFs (CAFs1–10). −ΔCT values are shown. (**D**) ELISA analysis of TGF-β1 levels in the CM of CAFs1–10.

To observe the direct effect of CAFs on tumor cell-intrinsic cGAS–STING expression in CRC, we established primary CAFs from CRC specimens and co-cultured them with CRC cell lines in vitro. CRC tissue specimens from each patient in the FMU Cohort 2 were dissociated using the gentleMACS Dissociator with the Tumor Dissociation Kit, human. The dissociated cells were then seeded in a type I collagen-coated 10 cm plate and cultured until the third passage (Fig. 2A), after which the cultured exhibited a fibroblast-like appearance (Fig. 2A), and > 85% of these cells were primary CAFs (CD45^–^EpCAM^–^CD31^–^CD90^+^ cells)^31,32^ as confirmed by flow cytometry (Fig. 2B). We established a total of 10 distinct primary CAFs from different CRC patients (Table 2). All CAFs expressed representative CAF markers, such as *VCAN*, periostin (*POSTN*), fibronectin 1 (*FN1*), and α-smooth muscle actin (*ACTA2*), and produced TGF-β1, but the expression levels of these markers varied among the 10 CAF lines (Fig. 2C, D). Primary CAFs were used in further experiments at the earliest passage, between the third and tenth passages, since their characteristics as primary CAF were confirmed at the earlier passage (Supplementary Fig. S1).

**Table 2:** Patient and tumor characteristics (FMU cohort 2).

No	Age, years	Sex	Location	Tumor depth invasion	LN metastasis	Distant metastasis	pStage	Pathology	MSI status
1	77	Female	Transverse colon	T3	Present	Absent	IIIb	tub2	MSS
2	53	Male	Rectum	T2	Absent	Absent	I	tub1 > pap	MSS
3	72	Male	Sigmoid colon	T4	Absent	Absent	IIb	tub2 > tub1	MSS
4	53	Male	Sigmoid colon	T3	Absent	Absent	IIa	tub2 > tub1	MSS
5	72	Male	Cecum	T4	Absent	Absent	IIb	tub2	MSS
6	77	Male	Proctos	T2	Absent	Absent	I	muc	MSS
7	73	Female	Sigmoid colon	T3	Present	Absent	IIIc	tub2 > muc	MSS
8	50	Male	Cecum	T2	Absent	Absent	I	tub1 > tub2 > pap	MSS
9	51	Male	Rectum	T2	Absent	Absent	I	tub2 > tub1	MSS
10	60	Male	Rectum	T2	Absent	Absent	I	tub1 > tub2	MSS

*FMU* Fukushima medical university, *LN* lymph node, *pStage* pathological stage, *MSI* microsatellite instability, *MSS* microsatellite stable.

**Fig. 3:**
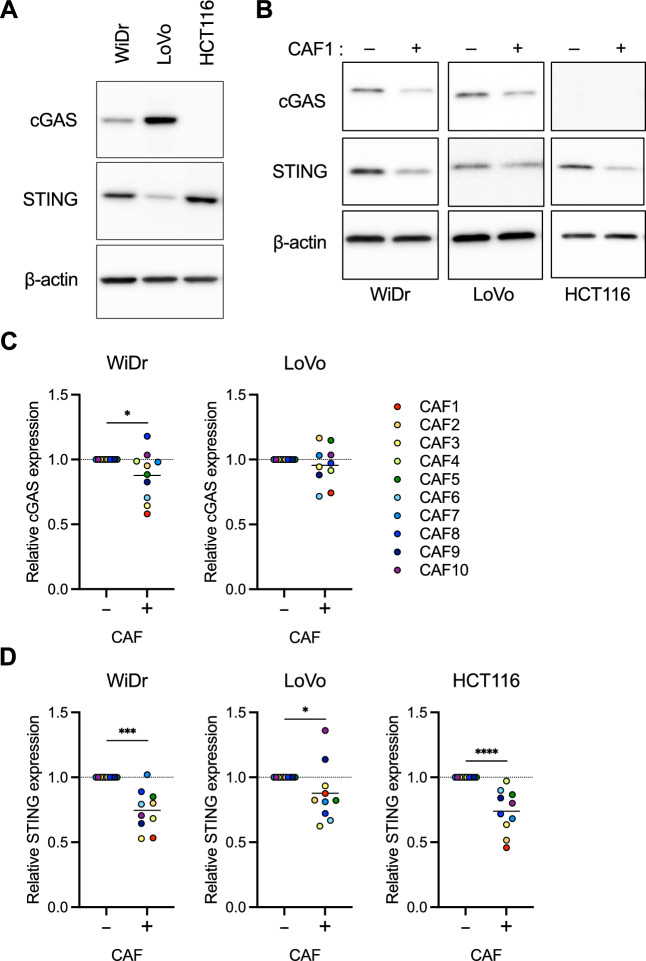
Downregulation of cGAS–STING expression in CRC cells by co-culture with CAFs. (**A**) Western blot analysis of cGAS and STING in WiDr, LoVo, and HCT116 cells, with β-actin as a loading control. (**B**) Western blot analysis of cGAS and STING in WiDr, LoVo, and HCT116 cells co-cultured with CAFs for 72 h. β-actin was used as a loading control. (**C, D**) The quantification of cGAS and STING was normalized to β-actin, presenting the relative expression levels of cGAS (**C**) and STING (**D**) in WiDr, LoVo, and HCT116 cells co-cultured with CAFs compared to the control (CRC cells alone). Values are presented as mean ± SD. Statistical significance was determined using the Mann–Whitney *U* test. *p < 0.05, ***p < 0.001, ****p < 0.0001.

Among the three CRC cell lines (WiDr, LoVo, and HCT116) used in the co-culture experiments with CAFs, Western blot analysis revealed that WiDr and LoVo cells expressed both cGAS and STING, whereas HCT116 cells expressed only STING (Fig. 3A). Intriguingly, co-culturing with CAFs using a 0.4 μm transwell insert decreased the expression of cGAS and/or STING in WiDr, LoVo, and HCT116 cells, as shown in the representative Western blot analysis (Fig. 3B). Statistical analysis of tumor cell-intrinsic cGAS and STING expression, co-cultured with CAFs derived from 10 different patients, demonstrated that CAFs significantly reduced cGAS expression in WiDr cells and markedly suppressed STING expression in WiDr, LoVo, and HCT116 cells (Fig. 3C, D). Quantitative analysis of cGAS expression in HCT116 cells could not be performed because cGAS expression was undetectable in Western blot analysis (Fig. 3C). In contrast, representative Western blot analysis of cGAS and STING in CRC cells co-cultured with normal fibroblasts (NFs) suggest that NFs might have a less pronounced effect on cGAS–STING expression in CRC cells compared to CAFs (Supplementary Fig. S2). Additionally, co-culture with CAFs decreased the levels of both phosphorylated IRF3 and phosphorylated signal transducer and activator of transcription 1 (STAT1), markers of cGAS–STING pathway activation, in WiDr cells (Supplementary Fig. S3). These findings suggest that CAFs can downregulate the tumor cell-intrinsic cGAS–STING expression, with a more pronounced suppressive effect on STING expression in CRC cells via certain secreted factors.

### Characterization of CAFs that negatively impact cGAS–STING expression in CRC cells

**Fig. 4:**
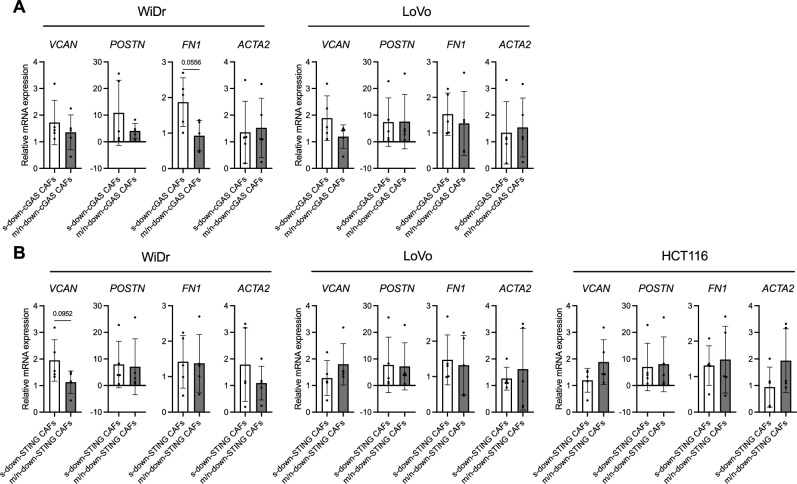
CAF markers highly expressed in CAFs that negatively impact cGAS–STING expression in CRC cells. (**A, B**) Comparison of CAF marker gene expression, as determined by qPCR, between CAFs that strongly downregulated cGAS (s-down-cGAS CAFs) and those that moderately/not downregulated cGAS (m/n-down-cGAS CAFs) (**A**), and between CAFs with strongly downregulated STING (s-down-STING CAFs) and those with moderately/not downregulated STING (m/n-down-STING CAFs) (**B**). Values are presented as mean ± SD. Statistical significance was determined using the Mann–Whitney *U* test.

To further characterize the CAFs that downregulate cGAS–STING expression in CRC cells, we compared the basal mRNA expression levels of CAF markers between CAFs that strongly downregulated cGAS–STING expression in CRC cells (s-down-cGAS/STING CAFs) and those that moderately or did not downregulate cGAS–STING expression (m/n-down-cGAS/STING CAFs), based on the median reduction of cGAS–STING expression in CRC cell lines. Since the CAFs that strongly downregulated cGAS or STING expression varied across CRC cell lines, we presented the expression patterns of CAF markers for each CRC cell line individually. *FN1* mRNA expression tended to be higher in s-down-cGAS CAFs compared to m/n-down-cGAS CAFs in WiDr cells (*p* = 0.0556) (Fig. 4A). Similarly, *VCAN* mRNA expression appeared higher in s-down-STING CAFs compared to m/n-down-STING CAFs in WiDr cells (*p* = 0.0952) (Fig. 4B), although there was no statistically significant difference among the samples.

TGF-β is one of the key factors secreted by CAFs^33^, and we confirmed that our established CAFs from CRC patients secreted a measurable amount of TGF-β (Fig. 2D). We, therefore, tested whether TGF-β1 could decrease the expression of cGAS–STING in CRC cells. However, treatment with recombinant human TGF-β1 (active form) did not affect cGAS–STING expression levels in WiDr, LoVo, or HCT116 cells (Supplementary Fig. S4), suggesting that TGF-β produced by CAFs might not be involved in the downregulation of cGAS–STING expression in CRC cells.

Taking these results, CAFs can downregulate tumor cell-intrinsic cGAS–STING expression in CRC. While CAFs expressing *VCAN* and *FN1* appear to mediate this downregulation, the precise characteristics of CAFs or the mediators responsible for this effect remain unclear.

## Discussion

To the best of our knowledge, the present study is the first to demonstrate that CAFs may be involved in the downregulation of tumor cell-intrinsic cGAS–STING expression in CRC. We found that stromal VCAN-high, immunosuppressive CAF-rich CRC exhibited lower tumor cell-intrinsic cGAS–STING expression compared to stromal VCAN-low CRC. Furthermore, our in vitro experiments revealed that co-culture with CAFs significantly decreased cGAS–STING expression, with a particularly strong inhibition of STING expression, in CRC cell lines. These findings suggest that CAFs in the TME may negatively impact tumor cell-intrinsic cGAS–STING expression in CRC, potentially suppressing anti-tumor immune responses.

The regulatory mechanism underlying the CAF-induced downregulation of tumor cell-intrinsic cGAS–STING expression in CRC in the present study remains unclear. CAFs produce and secrete various mediators, including cytokines, chemokines, growth factors, and extracellular matrix components^34^. Among these molecules, TGF-β is one of the representative factors^33^. However, treatment with an active form of recombinant human TGF-β did not reduce cGAS–STING expression in the CRC WiDr, LoVo, and HCT116 cell lines (Supplementary Fig. S4). TGF-β pathway mutations were not observed in LoVo and HCT116 cells^35^. Thus, TGF-β produced by CAFs might not be involved in the downregulation of cGAS–STING expression in CRC cells. Recent studies suggest that the activation of intracellular Akt signaling selectively abrogates STING signaling and its expression in several types of cancer cells, including CRC cells^36–41^. Therefore, secreted factors from CAFs related to Akt signaling activation might be involved in the downregulation of STING expression in the CRC cells. To identify the specific CAF-derived molecule responsible for the downregulation of cGAS–STING expression in CRC cells will be a focus of our future research.

Some previous studies have examined the role of the cGAS–STING pathway in CAFs and its positive effect on the activation of the anti-tumor immune response. Arwert et al. demonstrated that the transcytosis of cytoplasm from cancer cells into CAFs can activate the STING pathway in CAFs, particularly in tumors such as squamous cell carcinoma, thereby promoting anti-tumor immune responses^42^. Moreover, Suzuki et al. suggested that STING activation in CAFs enhances anti-tumor immunity in pancreatic cancer^43^. These findings suggest that CAFs may contribute to the anti-tumor immune response through the activation of their own STING signaling. Based on our IHC analysis of STING in CRC, we have identified four distinct groups within the CRC TME based on differences in STING expression between stromal areas and tumor cells: stromal STING^high^/tumor cell STING^high^, stromal STING^high^/tumor cell STING^low^, stromal STING^low^/tumor cell STING^high^, and stromal STING^low^/tumor cell STING^low^ CRCs (Supplementary Figure S5). In particular, in the stromal STING^low^ CRC group, the phenomenon described in the previous studies may not occur, and it is possible that CAFs in this subgroup contribute to the reduction of STING expression in tumor cells. However, this remains a hypothesis, and further detailed studies are needed to clarify the specific CRC TME conditions that lead to CAF-mediated negative regulation of STING expression in tumor cells.

In conclusion, CAFs can directly downregulate tumor cell-intrinsic cGAS–STING expression in CRC. Since downregulated tumor cell-intrinsic cGAS–STING expression is associated with decreased frequency of CD8^+^ TILs, targeting CAFs could represent a novel therapeutic strategy to improve patient outcomes in CRC.

## Methods

### CRC patient samples

We enrolled patients with CRC who had undergone surgical resection at FMU Hospital during two distinct periods: FMU Cohort 1 (2002–2013; *n* = 268) (Fig. 1 and Table 1) and FMU Cohort 2 (2024; *n* = 10) (Figs. 2, 3 and 4) (Table 2). Clinical and pathological data were retrospectively collected from medical records. This study was approved by the Research Ethics Committee of FMU (Approval Nos. 2289, 2847, and REC2024-041), and all procedures were conducted in accordance with the Declaration of Helsinki. Written informed consent was obtained from all participants.

### IHC

IHC was performed as previously described^18,29^ using formalin-fixed paraffin-embedded CRC specimens from patients in FMU Cohort 1 (*n* = 268). The primary antibodies used in this study included anti-cGAS monoclonal antibody (#79978, RRID:AB_2905508; Cell Signaling Technology, Danvers, MA; diluted 1:300), anti-STING monoclonal antibody (#13647, RRID:AB_2732796; Cell Signaling Technology; diluted 1:200), and anti-VCAN polyclonal antibody (HPA004726, RRID:AB_1080561; Prestige Antibodies^®^ Powered by Atlas Antibodies, Sigma–Aldrich, St. Louis, MO; diluted 1:500).

### Assessment of IHC

The evaluation of cGAS, STING, and VCAN was conducted as previously described^18,29^. Briefly, cGAS–STING expression in tumor cells was assessed using the H-score, which ranges from 0 to 300, independent of expression in the stromal area. The H-score is calculated by multiplying the intensity score (0–3+) by the percentage of stained cytoplasm (0–100%)^18^. Stromal VCAN expression was evaluated using the VCAN IHC score, which ranges from 0 to 4 and is calculated by combining the intensity score (0–2) and extent score (0–2). Tumors were classified into stromal VCAN-low (VCAN IHC score 0–2) or stromal VCAN-high (score 3 and 4) groups^29^. Tumor with loss of at least one MMR protein (MLH1, MSH2, MSH6, or PMS2) were defined as dMMR, while those with intact MMR protein expression were defined as pMMR^44^. IHC analyses of each molecule were evaluated by at least two observers, including R.K., A.K., S.C., and S.N., all of whom were blinded to the clinical and pathological data.

### Isolation of human primary NFs and CAFs from CRC tissues

CAF isolation was performed using fresh CRC tissue specimens from patients in FMU Cohort 2 (*n* = 10; patient Nos. 1–10), all of whom had not received preoperative chemotherapy. Similarly, NF isolation was conducted using fresh adjacent normal mucosa located at least 5 cm away from the cancer site in patients from FMU Cohort 2 (*n* = 2; patient Nos. 6 and 8). First, the specimens from normal mucosa and CRC tissues were rinsed with PBS containing 1% penicillin/streptomycin, cut into small pieces with scalpels, and dissociated using the gentleMACS^™^ Dissociator (130-093-235; Miltenyi Biotec, Bergisch Gladbach, Germany) with the Tumor Dissociation Kit, human (130-095-929; Miltenyi Biotec) at 37 °C for 1 h (program no. 37C_h_TDK_1). The collected cells were filtered using a 70 μm cell strainer, washed twice with RPMI-1640 medium, and then seeded into collagen-coated 10 cm dishes at approximately 3–5 × 10^6^ cells. The cells were cultured until the third passage to enrich NFs and CAFs.

### Flow cytometry analysis

CAFs were stained with various reagents and antibodies, including BD Horizon^™^ Fixable Viability Stain 780 (565388, RRID:AB_2869673; BD Biosciences, San Jose, CA; diluted 1:1000), PerCP/Cyanine5.5 anti-human CD326 (EpCAM) Antibody (324213, RRID:AB_893474; BioLegend, San Diego, CA), PE/Cyanine7 anti-human CD31 Antibody (303117, RRID:AB_2114314; BioLegend), Alexa Fluor^®^ 488 anti-human CD45 Antibody (304019, RRID:AB_493033; BioLegend), and Alexa Fluor^®^ 647 anti-human CD90 (Thy1) Antibody (328115, RRID:AB_893439; BioLegend). All antibodies were used at a 1:100 dilution. Unstained samples were used as negative controls. Flow cytometry analysis was performed using a BD FACS Canto II flow cytometer (BD Biosciences), and the data were analyzed with FlowJo software version 10.8.1 (RRID:SCR_008520; FlowJo, Ashland, OR).

### CRC cell lines

Three human CRC cell lines, authenticated and characterized by the suppliers through short tandem repeat analysis, were purchased from RIKEN Cell Bank (HCT116, RRID:CVCL_0291) and JCRB Cell Bank (LoVo, RRID:CVCL_0399; WiDr, RRID:CVCL_2760). The cells were maintained in RPMI-1640 medium (Merck Sigma-Aldrich, St. Louis, MO) containing 10% heat-inactivated FBS and 1% penicillin/streptomycin at 37 °C in an atmosphere of 5% CO_2_. The cells were passaged no more than 15 times before a new frozen aliquot of cells was used.

### Co-culturing CRC cell lines with CAFs

Each CRC cell line was seeded at a density of 1.0 × 10^5^ cells per well in a 6-well plate and incubated overnight in RPMI-1640 medium supplemented with 10% FBS. The CRC cell lines were then co-cultured with CAFs at a density of 1.0 × 10^5^ cells per well using 0.4 μm pore-sized insert well chambers (353090; CORNING, Corning, NY) for 72 h in RPMI-1640 supplemented with 1% FBS. After co-culturing, CRC cells were subjected to Western blot analysis to assess the expression levels of cGAS and STING.

### Western blot analysis

Western blot analysis was performed as previously described^18^. Briefly, CRC cells were lysed in RIPA buffer containing protease and phosphatase inhibitors. Lysates were mixed with SDS sample buffer containing dithiothreitol and boiled. Ten micrograms of protein were applied to SDS-PAGE and transferred to PVDF membranes. The membranes were blocked and then incubated overnight at 4 °C with the following primary antibodies: anti-cGAS mAb (#79978, RRID:AB_2905508; Cell Signaling Technology; diluted 1:1000), anti-STING mAb (#13647, RRID:AB_2732796; Cell Signaling Technology; diluted 1:1000), anti-phospho-IRF-3 mAb (#37829, RRID:AB_2799121; Cell Signaling Technology; diluted 1:1000), anti-phospho-Stat1 mAb (#8826, RRID:AB_2773718; Cell Signaling Technology; diluted 1:1000), and anti-β-actin mAb (sc-69879, RRID:AB_1119529; Santa Cruz Biotechnology, Dallas, TX; diluted 1:5000). The membranes were subsequently incubated with HRP-conjugated secondary antibodies for 1 h at room temperature. Immunoreactive proteins were visualized using the ImageQuant LAS 4000 mini (Fuji Film, Tokyo, Japan) with ECL Prime Western Blotting Detection Reagent (Cytiva, Tokyo, Japan).

The original, unprocessed blot images are shown in Supplementary Fig. S6. The membranes were appropriately cut at specific positions before antibody incubation to ensure that each section was used for the intended antibody reactions. The specificity of cGAS and STING antibodies has been previously validated in our published studies^18,39,45–47^. Additionally, for Fig. 3A, we have provided the full membrane of the original blot in Supplementary Fig. S6, demonstrating that non-specific bands were minimal. Based on these observations, in subsequent experiments, we cut the membrane at 50 kDa before antibody incubation for cGAS and STING detection.

To ensure that all blots clearly display membrane edges, we have included images with enhanced contrast at the edges of the original blots. Capturing the membrane edge for β-actin (loading control) is difficult due to the high sensitivity of the antibody, and a short exposure time was sufficient to obtain a clear band. We used the same blot for both STING and β-actin detection, and the β-actin band size was confirmed using the LAS4000 imaging system when capturing the blot images.

### Reverse transcription-quantitative PCR

Total RNA was isolated from CAFs using TRIzol Reagent (QIAGEN, Valencia, CA). The extracted RNA was quantified using a NanoDrop ND-1000 spectrophotometer (Thermo Fisher Scientific, Waltham, MA). Reverse transcription-quantitative PCR (RT-qPCR) was performed on a QuantStudio 3 real-time PCR system (Applied Biosystems, Carlsbad, CA) using PrimeTime One-Step 4 × RT-qPCR Master Mix (10011744; Integrated DNA Technologies, Coralville, IA) along with specific primers and probes for *VCAN* (Accession No: NM_004385), *FN1* (Accession No: NM_212482), *ACTA2* (Accession No: NM_001613), *POSTN* (Accession No: NM_006475), and *ACTB* (Accession No: NM_001101) (Integrated DNA Technologies). Gene expression levels were normalized to *ACTB*.

### Enzyme-linked immunosorbent assay of CAF conditioned medium

CAFs were seeded into collagen-coated 10 cm dishes and cultured until they reached 90% confluence, after which the cells were washed twice with PBS, and 10 ml of serum-free RPMI-1640 medium was added. The cells were then cultured for an additional 48 h. The CM was collected, centrifuged at 1500 rpm for 5 min at 4 °C, and stored at − 80 °C until use in enzyme-linked immunosorbent assay (ELISA). The concentration of human TGF-β1 in the CAF CM was determined using the Human/Mouse/Rat/Porcine/Canine TGF-beta 1 Quantikine ELISA Kit (DB100C; R&D Systems, Minneapolis, MN) according to the manufacturer’s protocol.

### Statistical analysis

Data are expressed as means ± SD. Statistical analyses were carried out using GraphPad Prism 9 version 9.5.1 (RRID:SCR_002798; GraphPad Software, San Diego, CA). The Mann–Whitney *U* test was employed for two-group comparisons of means. Categorical variables were compared using either Fisher’s exact test or the Chi-square test. A *p* value of less than 0.05 was considered to indicate statistical significance.

## Supplementary Information

Supplementary Information.

## Data Availability

The datasets generated and/or analyzed during this study are available from the corresponding author upon reasonable request.

## Abbreviations

CAF: Cancer-associated fibroblast
cGAS: Cyclic GMP–AMP synthase
CM: Conditioned medium
CRC: Colorectal cancer
STING: Stimulator of interferon genes
*FN1*: Fibronectin 1
IFN: Interferon
M2-TAMs: M2-tumor-associated macrophages
MSI: Microsatellite instability
MMR: Mismatch repair
TGF-β: Transforming growth factor-β
TILs: Tumor-infiltrating lymphocytes
TME: Tumor microenvironment
