# Supplementary material for: Downregulation of cGAS/STING expression in tumor cells by cancer-associated fibroblasts in colorectal cancer
Source: Sci Rep. 2025 Jun 2;15:19234. doi: 10.1038/s41598-025-03924-6 (PMC12127464; doi:10.1038/s41598-025-03924-6)
Supplement: Supplementary file 1 — Supplementary Information. [file 41598_2025_3924_MOESM1_ESM.pdf]

Supplementary Figure S1

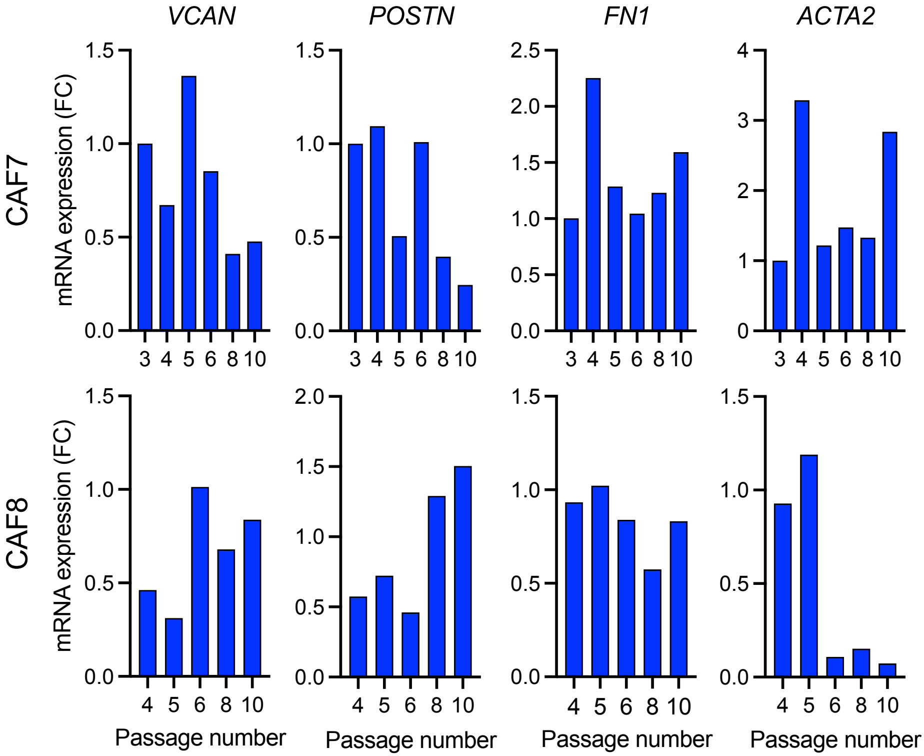

Supplementary Figure S1. Passage number-dependent expression of CAF markers in CAFs. qPCR analyses of the indicated molecules in CAF7 and CAF8 in a passage number-dependent manner. The x-axis represents passage numbers.

Supplementary Figure S2

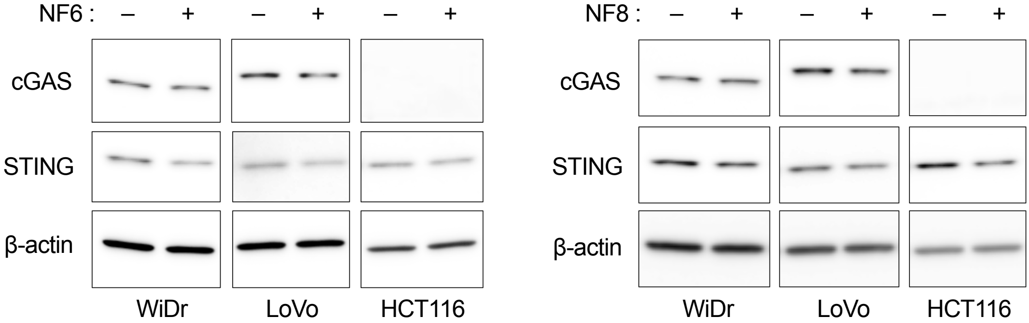

Supplementary Figure S2. The effect of NFs on cGAS–STING expression in CRC cells. Western blot analysis of cGAS and STING in WiDr, LoVo, and HCT116 cells co-cultured with NFs for 72 hours. β-actin was used as a loading control.

Supplementary Figure S3

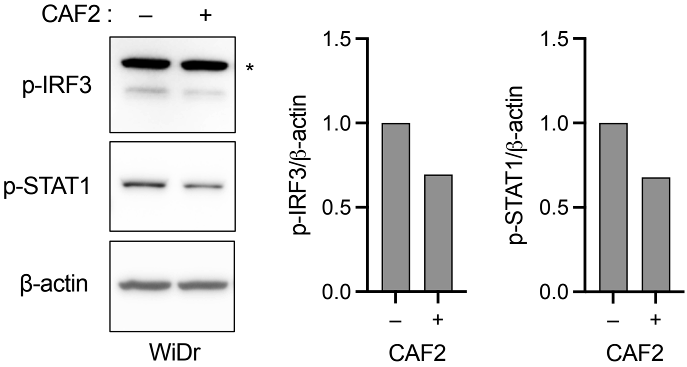

Supplementary Figure S3. Downregulation of cGAS–STING pathway activation by CAFs in CRC cells. Western blot analysis of phosphorylated IRF3 (p-IRF3) and phosphorylated STAT1 (p-STAT1) in WiDr cells co-cultured with CAF2 for 72 hours. β-actin was used as a loading control. The quantification of p-IRF3 and p-STAT1 was normalized to β-actin, presenting the relative expression levels of p-IRF3 and p-STAT1 in WiDr cells co-cultured with CAF2 compared to the control (WiDr cells alone). *Non-specific bands are indicated.

Supplementary Figure S4

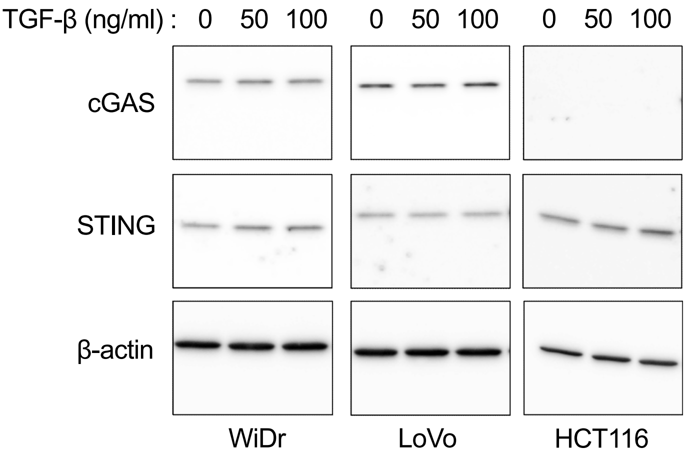

Supplementary Figure S4. The effect of TGF-β on cGAS–STING expression in CRC cells. Western blot analysis of cGAS and STING in WiDr, LoVo, and HCT116 cells treated with 0–100 ng/ml of active recombinant human TGF-β for 72 hours. β-actin was used as a loading control.

Supplementary Figure S5

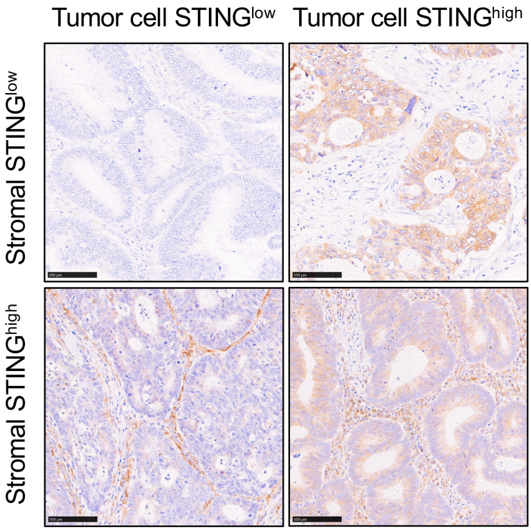

Supplementary Figure S5. Four distinct CRC groups were identified based on differences in STING expression between stromal areas and tumor cells: stromal STING^{high}/tumor cell STING^{high}, stromal STING^{high}/tumor cell STING^{low}, stromal STING^{low}/tumor cell STING^{high}, and stromal STING^{low}/tumor cell STING^{low} CRCs. Scale bars: 100 μm.

Supplementary Figure S6

Fig. 3A

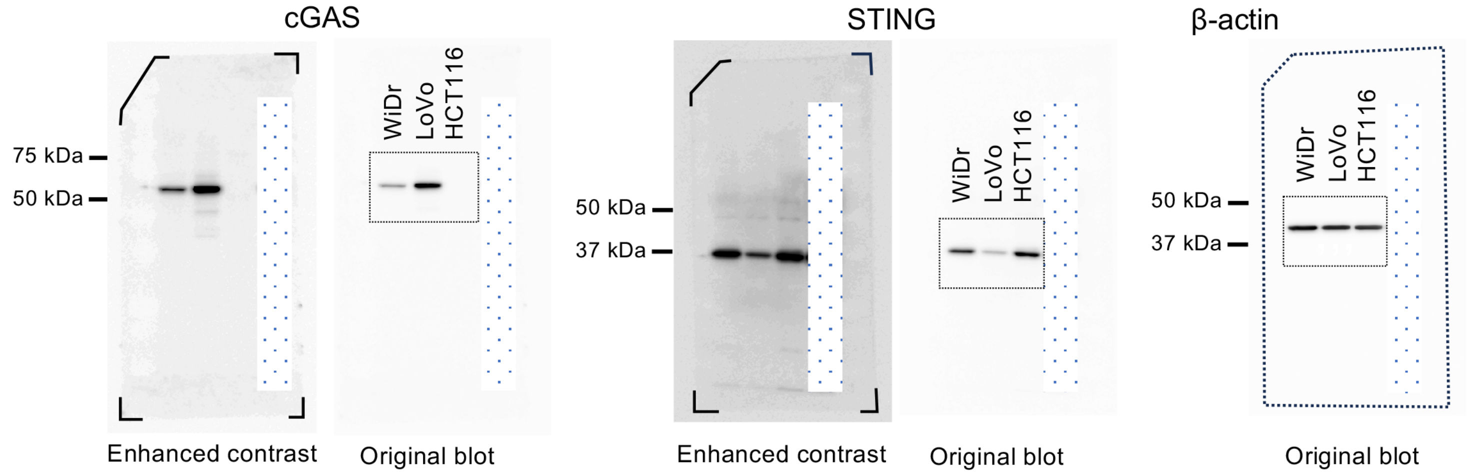

Fig. 3B

CAF1

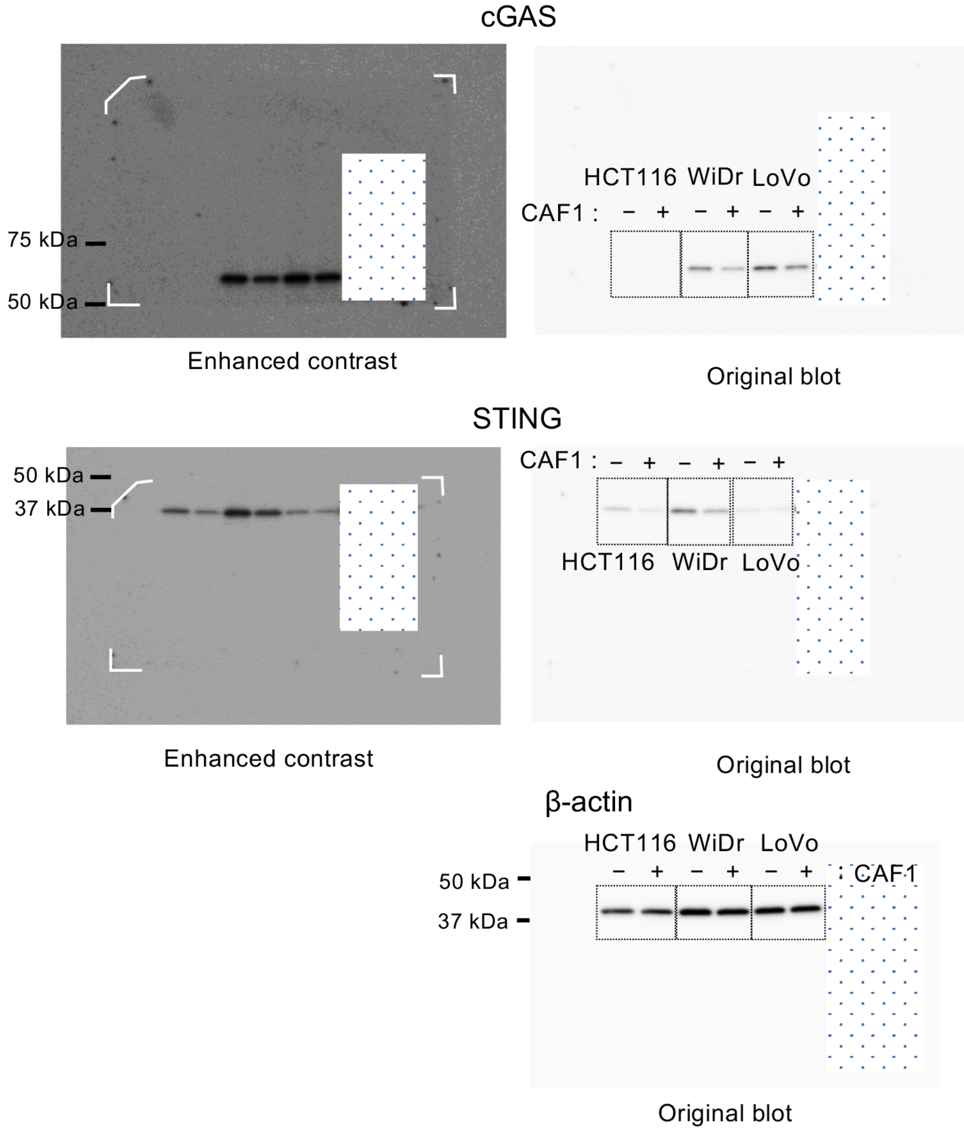

Supplementary Figure S6. Unprocessed scans of the original blots shown in figures and supplementary figures.

Supplementary Figure S6 Continued

Fig. 3B

CAF2

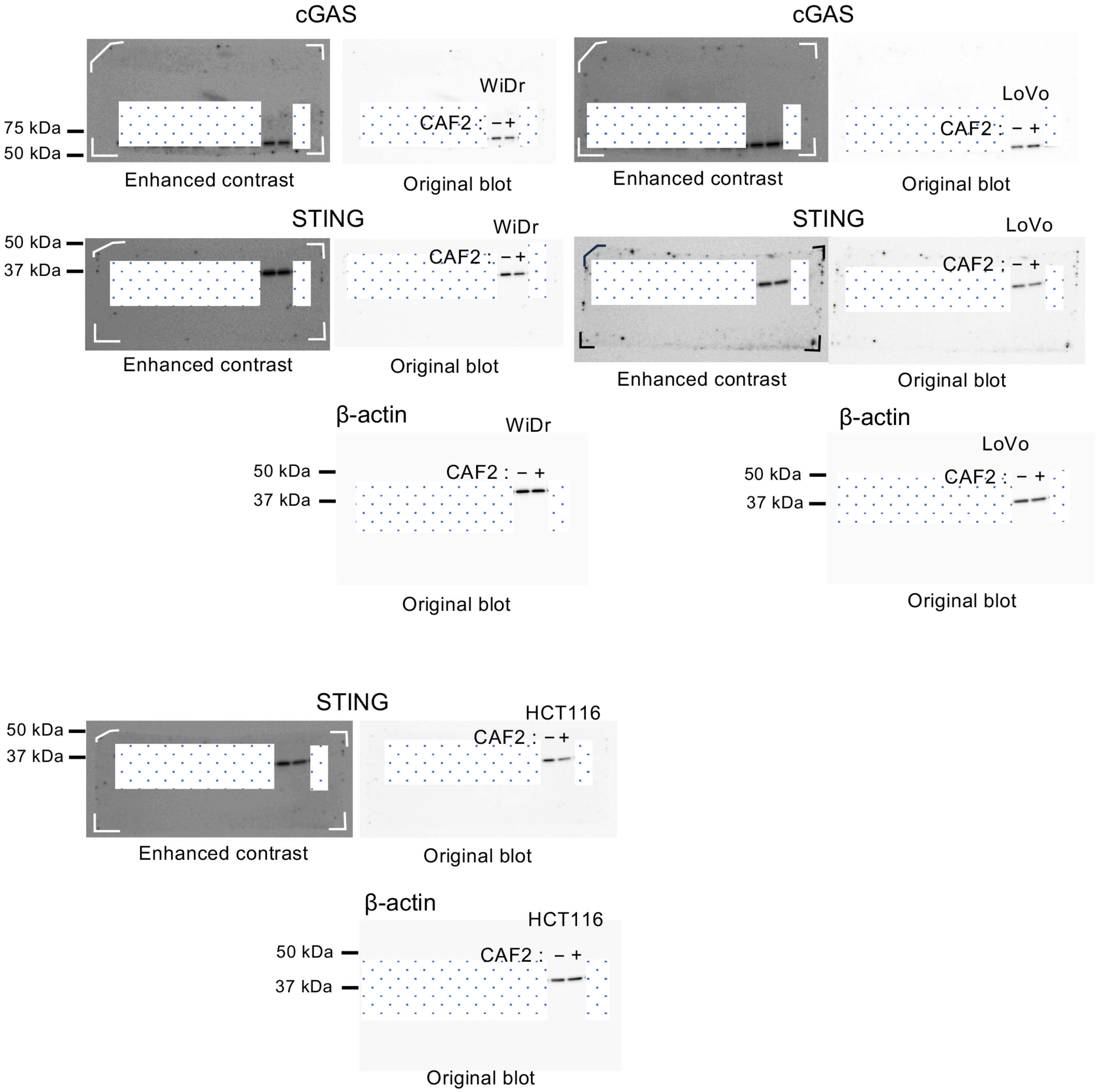

Supplementary Figure S6 Continued

Fig. 3B

CAF3

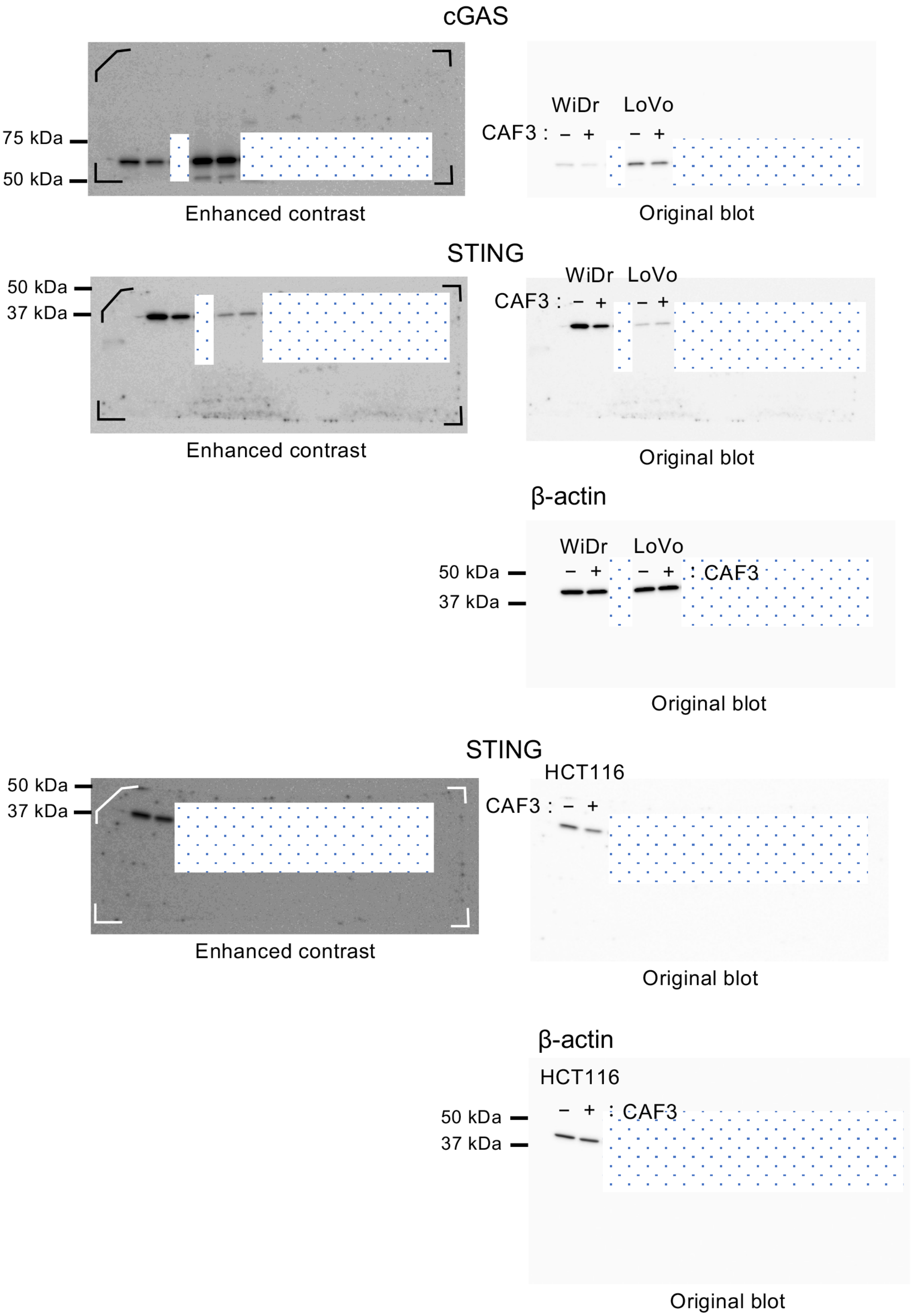

Supplementary Figure S6 Continued

Fig. 3B

CAF4

cGAS

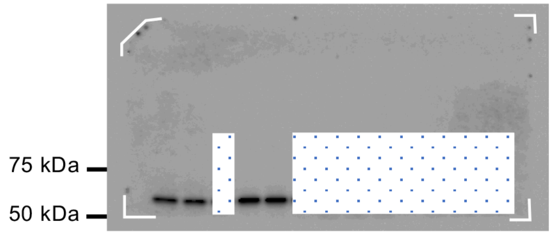

Enhanced contrast

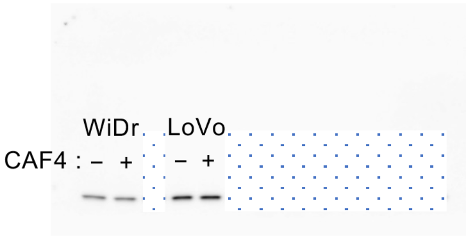

Original blot

STING

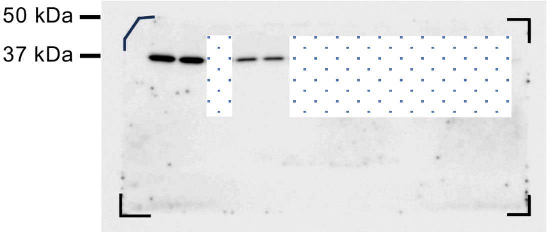

Enhanced contrast

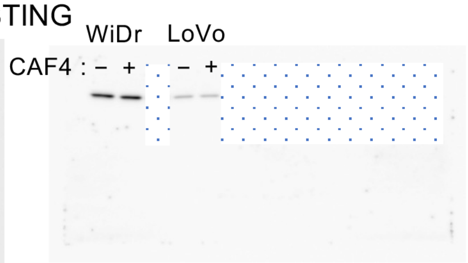

Original blot

β-actin

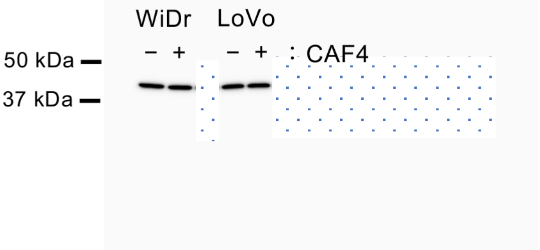

Original blot

STING

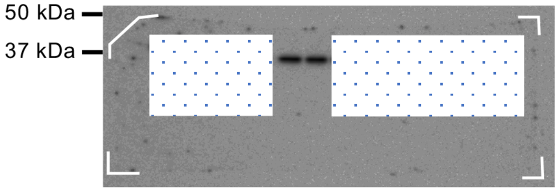

Enhanced contrast

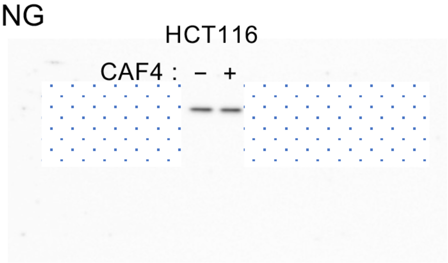

Original blot

β-actin

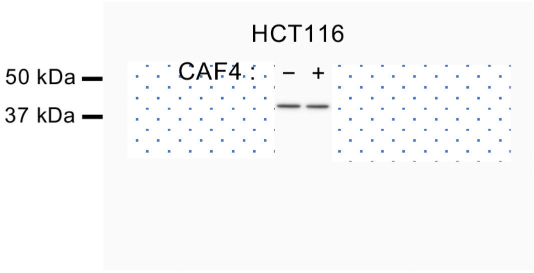

Original blot

Supplementary Figure S6 Continued

Fig. 3B

CAF5

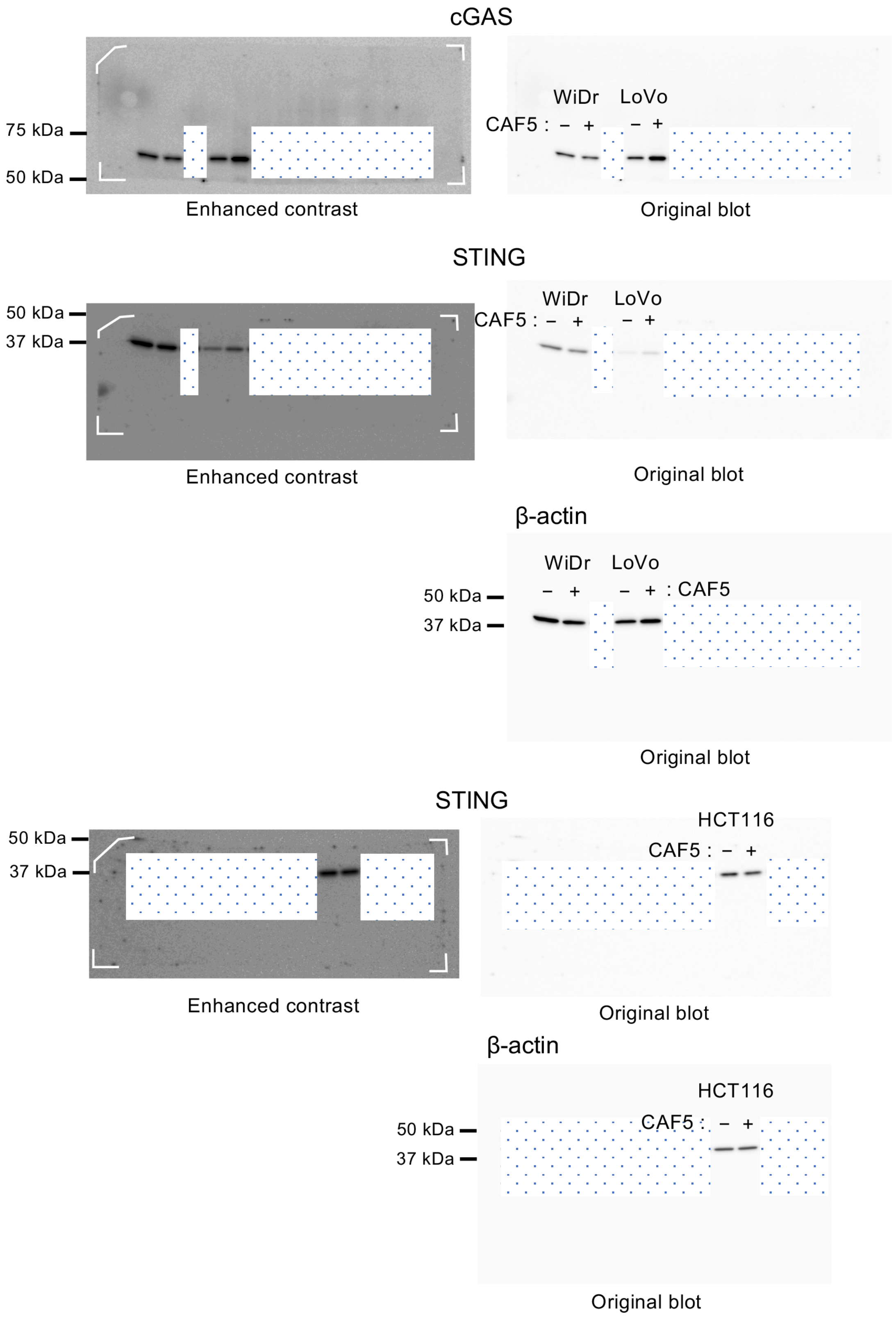

Supplementary Figure S6 Continued

Fig. 3B

CAF6

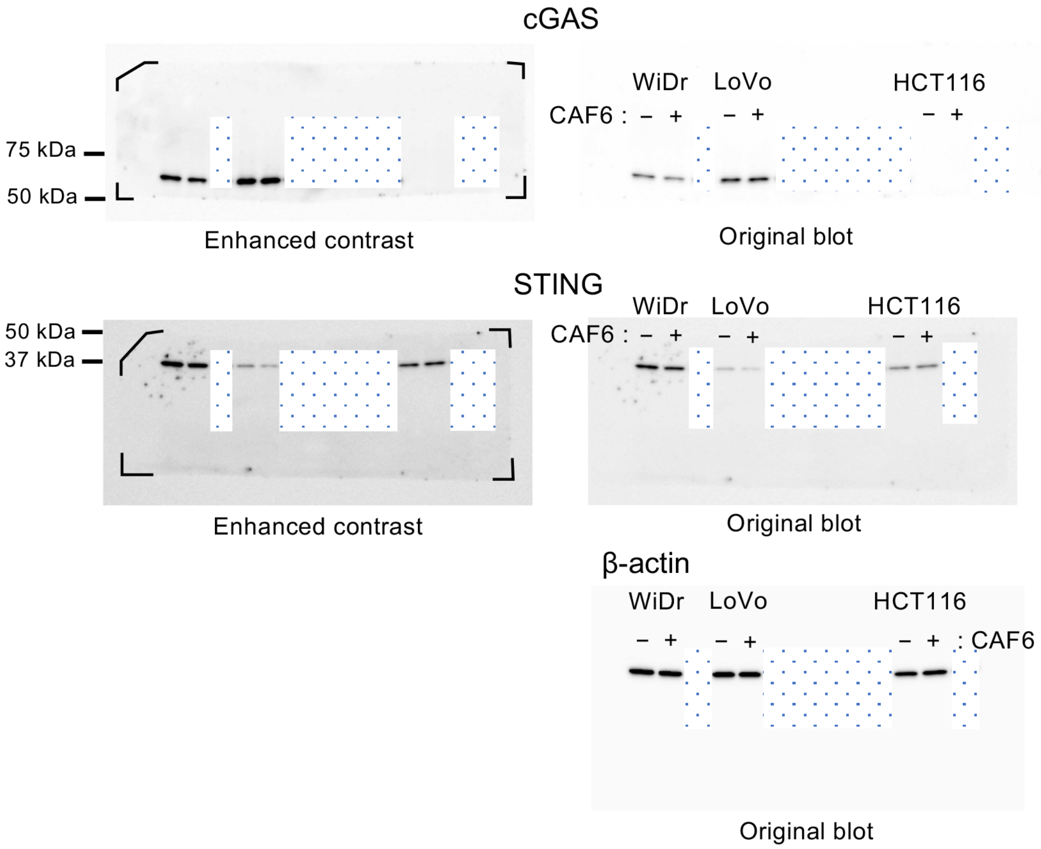

CAF7

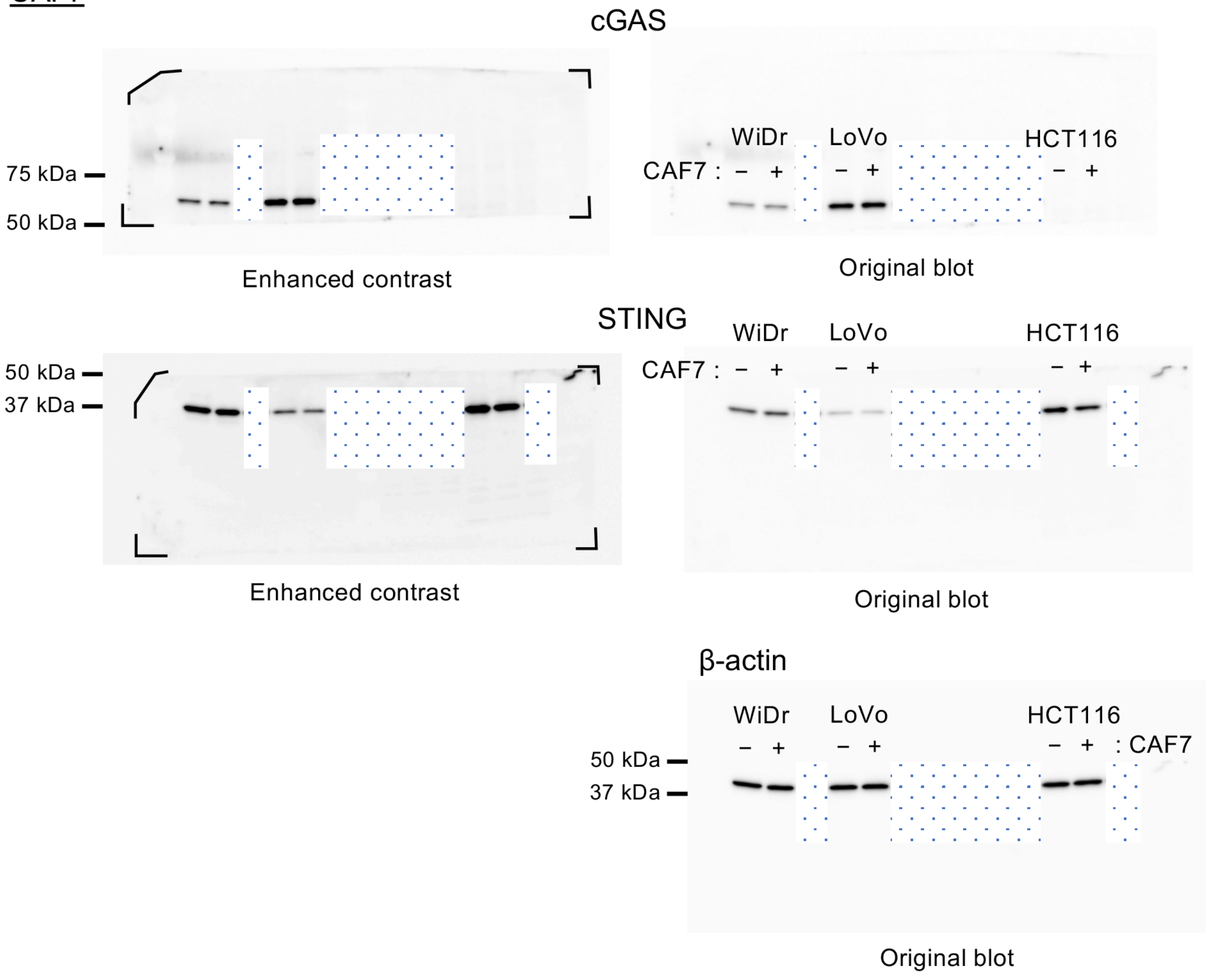

Supplementary Figure S6 Continued

Fig. 3B

CAF8

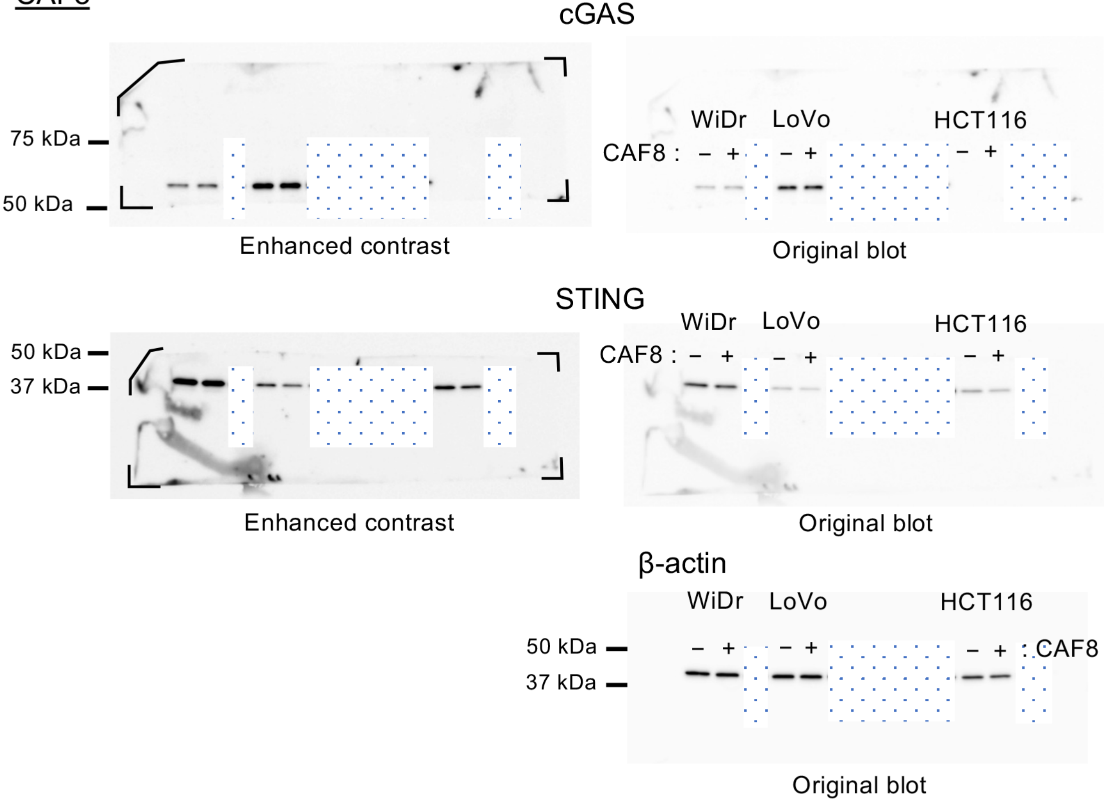

CAF9

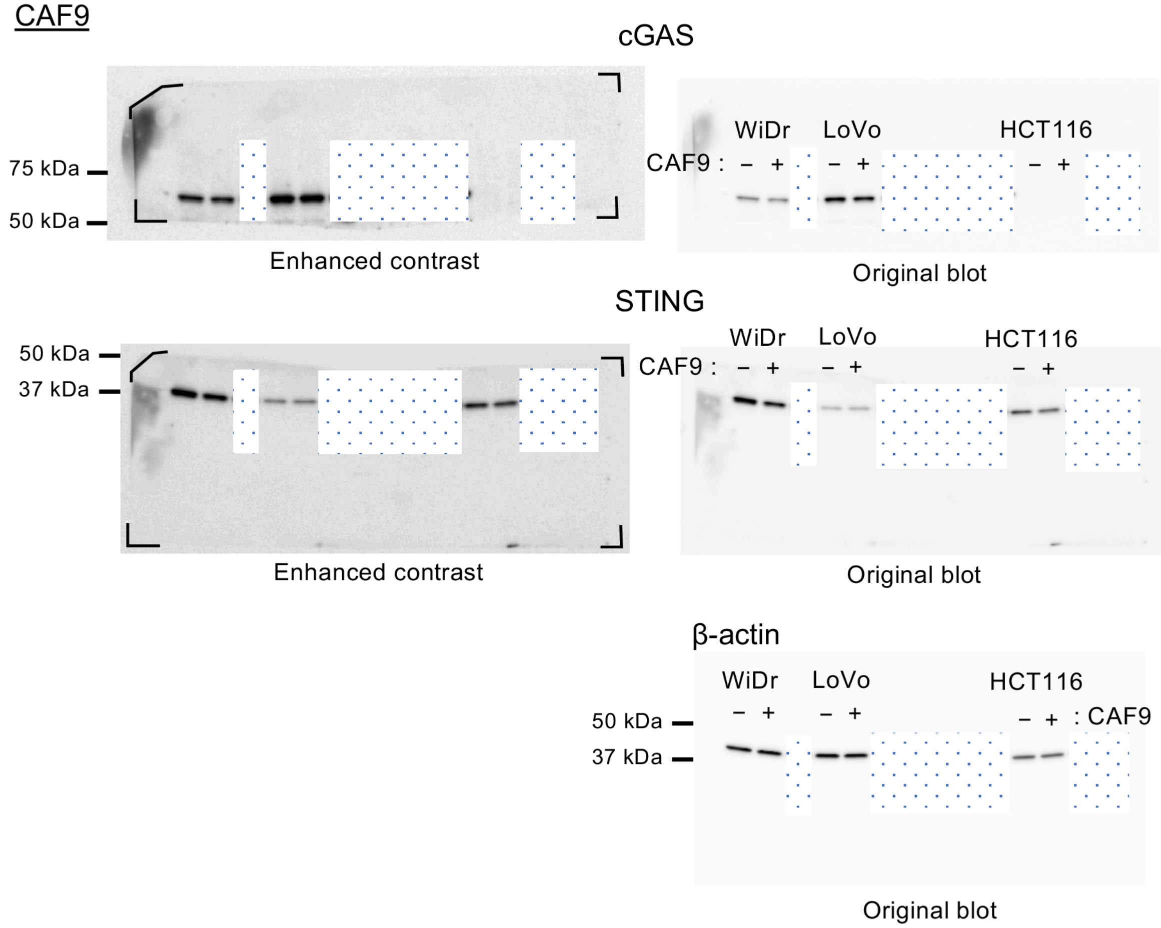

Supplementary Figure S6 Continued

Fig. 3B

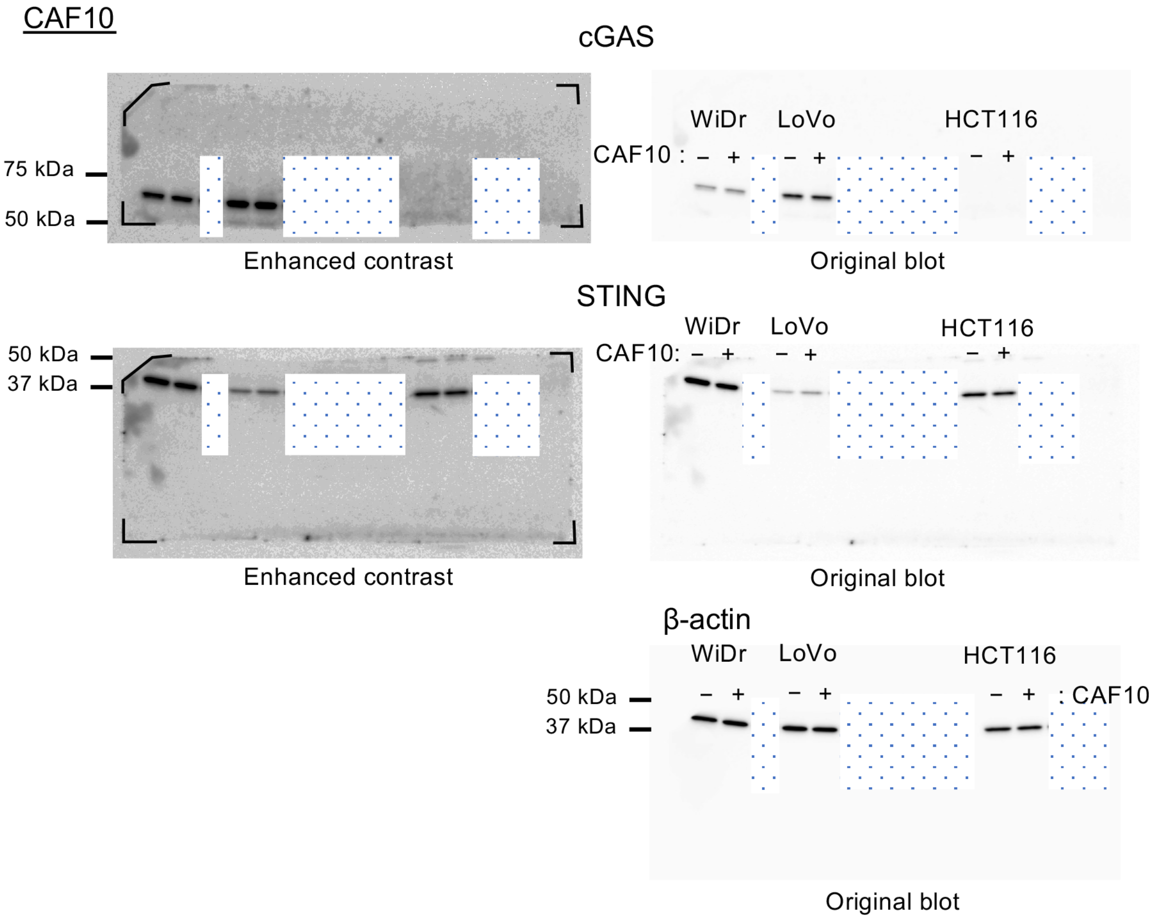

Supplementary Figure S6 Continued

Supplementary Fig. S2

NF6

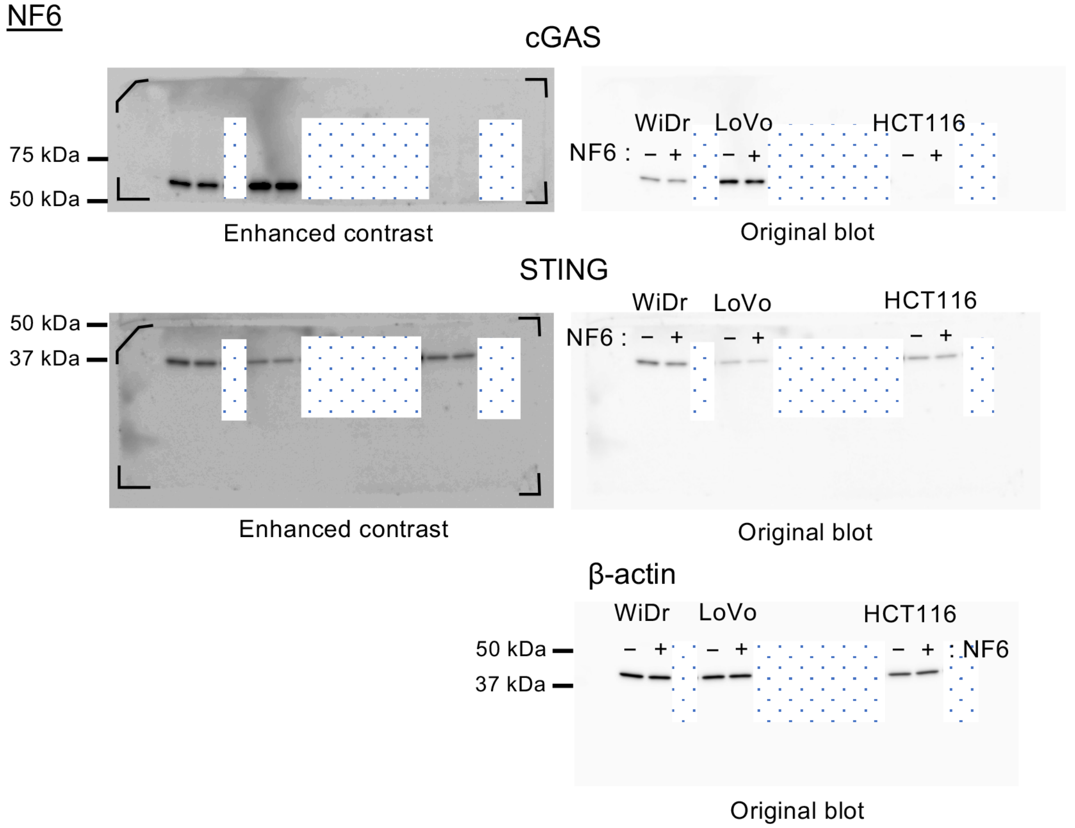

NF8

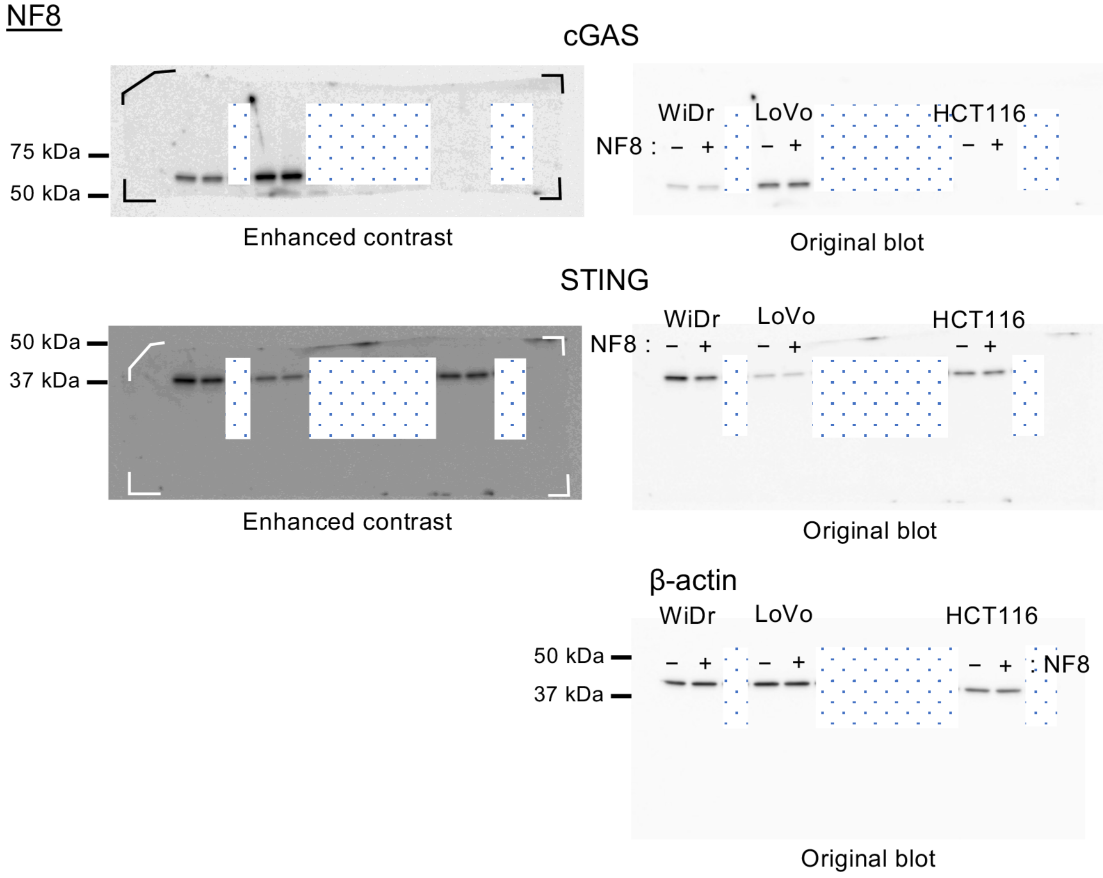

Supplementary Figure S6 Continued

Supplementary Fig. S3

p-IRF3

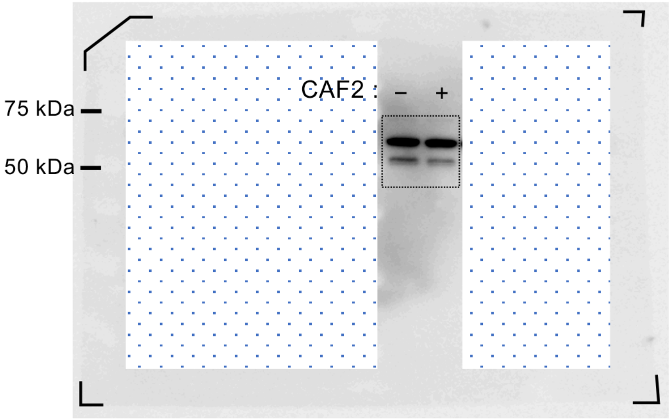

Enhanced contrast

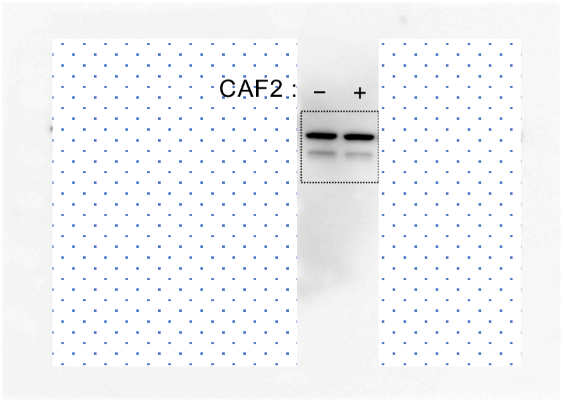

Original blot

p-STAT1

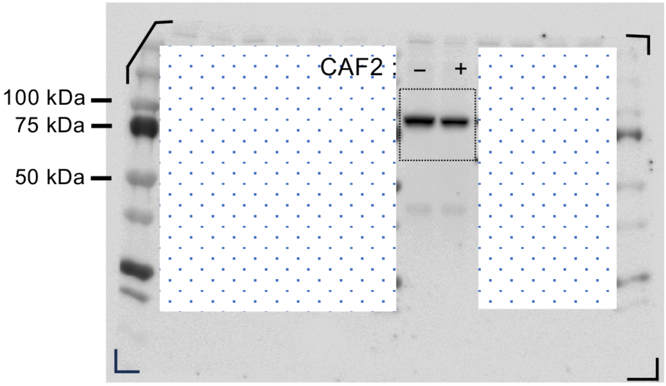

Enhanced contrast

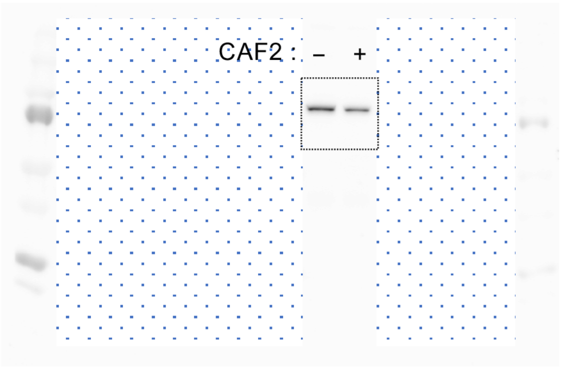

Original blot

β-actin

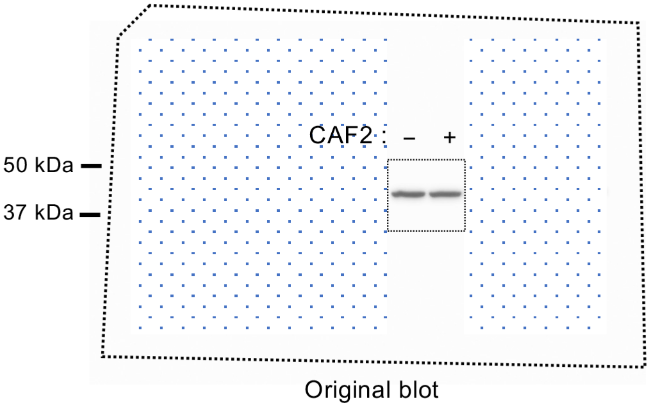

Original blot

Supplementary Figure S6 Continued

Supplementary Fig. S4

cGAS

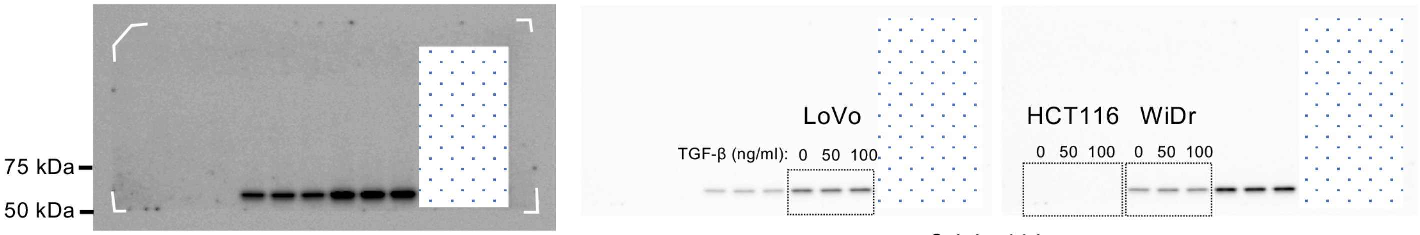

Enhanced contrast

Original blots

STING

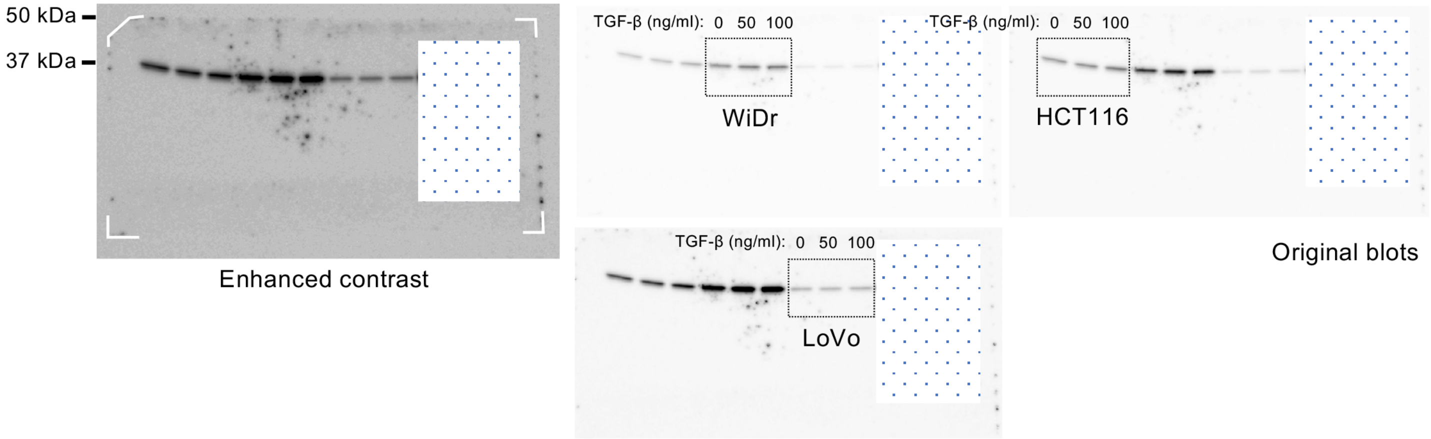

Enhanced contrast

Original blots

β-actin

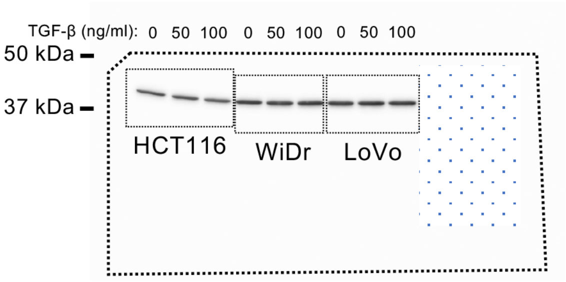

Original blot
